# Prescription of medicines by medical students of Karachi, Pakistan: A cross-sectional study

**DOI:** 10.1186/1471-2458-8-162

**Published:** 2008-05-19

**Authors:** Syed Nabeel Zafar, Reema Syed, Sana Waqar, Faria A Irani, Sarah Saleem

**Affiliations:** 1 Medical College, Aga Khan University, Stadium Road, Karachi, Pakistan; 2 Department of Community Health Sciences, Aga Khan University, Stadium Road, Karachi, Pakistan

## Abstract

**Background:**

Prescription of medicines by non-doctors is an issue with serious global implications. To our knowledge prescription of drugs by medical and non-medical students has not been studied before. We aimed to determine the practice and attitudes of drug prescription by medical students and: a) how non-medical students respond to this practice, b) How this compares with the attitudes and practices of non-medical students.

**Methods:**

A cross-sectional study was conducted on a sample of 600 students randomly selected from 2 medical and 2 non-medical universities. Ethical requirements were ensured and data was collected using self administered questionnaires. The Chi square tests and logistic univariate regression analyses were performed using SPSS v 14 to identify associations and differences.

**Results:**

A total of 572 forms were completed and the sample consisted of 295 medical students and 277 non-medical students with no significant difference in their demographic profile. Of the 295 medical students 163 (55.3%) had prescribed a medicine independently and most (48.5%) said that they did this 2–3 times a year. The commonest reasons for this were 'previous experience' (68.7%), 'problem too trivial' (34.4%) and 'we knew everything about the condition' (31.3%). One-third (33.6%) of the undergraduate medical students thought that it was alright to independently diagnose an illness while a vast majority (78.3%) thought that it was alright for them to prescribe medicines to others. Common prescriptions were pain-killers, antipyretics, antiallergics and antibiotics. Medical students who prescribed medicines were of lesser age (CI = 1.366–1.887) and more likely to belong to the 1^st ^(CI = 3.588–21.731), 2^nd ^(CI = 2.059– 10.869) or 3^rd ^(CI = 4.331–26.374) year of medical college. One-third (33.9%) of the non-medical students reported that a medical student had prescribed medicines to them and 21.3% said that they trusted medical students and would follow their advice blindly. Many students thought it alright for medical students to diagnose and treat illnesses. A similar proportion of non-medical students (58.5%) reported prescribing medicines to others.

**Conclusion:**

Prescription of medicines by non-doctors is rampant and urgent corrective measures are warranted. We have highlighted areas for future research and intervention and have given a few recommendations.

## Background

Medicines are complex chemicals with many actions, many of which are besides the desired ones. They can interact with other drugs, interfere with normal bodily functions, enhance or suppress various enzymes and can have a multitude of adverse effects [1]. A doctor only after having undergone extensive training in human physiology, pathology, pharmacology and other critical subjects becomes apt enough to assess the benefits and risks of the situation and then prescribe a suitable medication. Therefore, drug prescription has been reserved only for these qualified, graduated doctors [2]. In some countries especially developing countries where resources are limited, health workers other than doctors have been given the power to prescribe drugs and other health care services to others [3]. However, these people have been trained appropriately and are licensed to do so. Every other person who prescribes a drug/s is not only acting outside the law but also doing something very perilous which can result in devastating effects for not only the individual but the society as a whole.

Unfortunately this is not well understood by many and the ease of acquiring medicines from a pharmacy without a prescription coupled with rigorous advertising by pharmaceutical companies empowers even the common man to take and to prescribe medicines to others with much ease [4]. People often rely upon informal drug distribution channels [5]. In Pakistan, like many other developing countries almost every pharmacy would sell a drug to a customer without even asking for a prescription [6]. Many pharmacists even take the next step and treat illnesses on their own accord. It has been seen that they usually do not prescribe the appropriate medicine [7]. In a country where it is so easy to 'act like a doctor' it makes sense that even medical students would indulge in this activity. This is an issue of great international significance as it leads to a multitude of problems including the emergence of multi-drug resistant organisms. In this era of frequent and easy cross continent travel, drug resistance developing in one area can rapidly spread to other parts of the world. Local measures taken to prevent this problem taken in such parts, no matter how thorough, would then come to naught due to these outside influences. More so, contagious diseases can remain perilously undetectable for long periods of time if a person harboring a deadly disease has been taking medicines for symptomatic relief without proper evaluation. Unhindered travel by such people can lead to devastating epidemics and pandemics.

It has been observed that medical students in our part of the world start prescribing medicines to each other and to other people much before they graduate from medical college. These observations however, to the best of our knowledge, have never been reported. However, it has been studied that even senior medical students are not apt enough to prescribe medicines, they are hesitant and their choice of medicine is inappropriate most of the time [2,8]. We therefore deemed it necessary to explore this issue in order for it to be addressed. We were of the opinion that many medical students prescribe medicines to others and we thought it necessary to find out exactly what proportions of them do this, why they do it and what their attitude is towards this. We hypothesized that medical students prescribe more medicines than non-medical students of the same age and background. We also thought it necessary to explore how seriously the advice of medical students is taken by others. The objectives of our study were as follows:

(i) To determine the attitude and practices of drug prescription by medical students.

(ii) To determine whether non-medical students take the advice of medical students seriously and

(iii) To compare the practice and attitudes of drug prescription by medical students with that of non-medical students belonging to the same background.

Much research is needed in order to address the issue of irrational drug use; the types, the amounts and the reasons must be explored [5]. We found many astonishing findings and even though our hypothesis proved to be wrong, it highlighted a serious problem which could lead to devastating global consequences if left unaddressed.

## Methods

We conducted a cross-sectional study on a population of 600 students from four universities in Karachi, which is the largest city of Pakistan with a population of over 13 million. Of all the universities in Karachi we randomly selected 2 medical universities and 2 non-medical ones. Weighted samples were taken from each institution according to the number of students enrolled in that particular university. A total sample of 300 medical students were taken from the two medical universities in the ratio of 2:1 (i.e. 200 from the institution where the 'total number of students' ≈ 1100 and 100 from the institution where 'total number of students' ≈ 550). Similarly a total of 300 non-medical students were taken from the two non-medical universities (a school of business studies and a school of arts) also in the ratio of 2:1 ('total number of students' ≈1000 and 500 respectively).

We took a convenience sample by approaching students sitting in the main courtyards and common rooms of these institutions during the second week of February 2007 (one day for each institution). The exclusion criteria was people not enrolled in that particular university and those who were not Pakistani by nationality, no such person was encountered and therefore no one was excluded from the study.

Data was collected via a self reported questionnaire which the authors had designed themselves (Additional file 1). There were separate questionnaires for medical students and non-medical students. Both of these were pre-tested on a group of 15 students respectively, prior to the study, no major changes were required and the results of this pre-test were discarded. Each questionnaire contained three parts: the first part which was the same in both the questionnaires dealt with basic demographic details of the participants, the second part dealt with the practice of drug prescription by both medical and non-medical students. For non-medical students this part also inquired about the receipt of prescriptions by medical students. The third part assessed the attitude of the participants in this regard.

Written informed consent was taken from each participant before administering the questionnaire and after explaining the purpose of the study. It was explained to them that they had no obligation to complete the questionnaire and could abandon it at any point without stating a reason. Confidentiality was maintained. No information that can link any individual to the data was recorded. This study had been ethically approved by the Department of Community Health Sciences, Aga Khan University Karachi. Approval was also taken from each institute before conducting the study.

Data was double entered on Epi-data version 3.1, was managed and analyzed on SPSS version 14. Descriptive analyses were performed and the results were tabulated where necessary. Differences in the demographic details of both groups were tested by using the Chi square test. Associations between demographic variables such as gender, age and year of study were tested with the practice of independently prescribing medicines using univariate logistic regression analyses. Differences between prescription by medical and non-medical students were also calculated using this test. The odds ratio (OR) and confidence intervals (CIs) of these associations were thus calculated.

## Results

With a response rate of 95.3%, 572 students participated in this study. There were 295 medical students and 277 non-medical students. The commonest reason for refusal to participate was lack of time. There was no significant difference in the demographic details of both the groups. There were a total of 235 (41.1%) males and 337 (58.9%) females. Majority of the participants were Muslims (94.5%) and the mean age was 21 years with a standard deviation of 1.8.

It was found that of the 295 medical students, 163 (55.3%) of them had prescribed medicines to some one else in the absence of the supervision of a certified medical practitioner. The most common reasons for this were 'previous experience' (68.7%), 'the problem was too trivial to go to a doctor' (34.4%) and 'we knew everything about the condition' (31.3%). This is presented in table 1. The medicines prescribed are shown in table 2. Pain killers (85.9%), antipyretics (66.9%), anti-allergics (42.3%) and antibiotics (38%) were most commonly given. 48.5% of these medical students reported that they prescribe these medicines 2–3 times a year, while 30.7% said that they do it every few months. Majority (77.6%) of these medical students said that the person seeking advice had asked them to prescribe medicines for him/her while a few (22.4%) admitted that they had volunteered to prescribe the medications.

**Table 1:** Why students independently prescribed medicines.

**Reason**	Medical Student* n (%)	**Non Medical Student^**# **^n (%)**
**Have had a good previous experience with the drug**	112 (68.7)	143 (88.3)
**The problem was too trivial to go to a doctor**	56 (34.4)	58 (35.8)
**I knew everything about the illness**	51 (31.3)	16 (9.9)
**Urgency of the situation**	27 (16.6)	38 (23.5)
**I got enough information from the media**	12 (7.4)	17 (10.5)
**Cost of consultation is too high**	4 (2.3)	9 (5.6)

* n = 163 # n = 162

**Table 2:** Medicines prescribed by students

**Medicine**	Medical students* n (%)	**Non Medical Students^**# **^n (%)**
**Pain killers**	140 (85.9)	138 (85.2)
**Anti-pyretics**	109 (66.9)	76 (46.9)
**Anti- Allergics**	69 (42.3)	69 (42.6)
**Anti biotics**	62 (38.0)	24 (14.8)
**Vitamins**	71 (43.6)	52 (32.1)
**Antacids**	22 (13.5)	34 (21.0)
**Tonics**	15 (9.2)	4 (2.5)
**Sleeping pills**	12 (7.4)	10 (6.2)
**Birth control pills**	11 (6.7)	10 (6.2)
**Herbal/Homeopathic**	10 (6.1)	11 (6.8)

* n = 163 # n = 162

Almost one out of every two (43.1%) medical student thought that it was ok for them to prescribe medicines for any condition while a vast majority (78.3%) thought that it was ok for a mild condition. One out of three (33.6%) medical students thought that it was ok for them to diagnose a disease in the absence of a certified medical practitioner while one out of every six (15.3%) thought that it was alright for them to treat an illness without any supervision.

Medical students who prescribed medicines to others were of lesser age (OR = 1.61, CI = 1.366–1.887) and more likely to belong to the 1^st ^(OR = 8.83, CI = 3.588–21.731), 2^nd ^(OR = 4.73, CI = 2.059–10.869) or 3^rd ^(OR = 10.68, CI = 4.331–26.374) year of medical college.

When non-medical students were questioned about referring to medical students for advice regarding their illness, 155 (56%) reported that they regularly consulted medical students while less than half (44.2%) of these consulted a certified doctor afterwards. One out of every three (33.9%) students reported that a medical student had prescribed medicines to them and most (72.3%) had requested the prescription. While 21.3% (n = 59) of the students said that they trusted medical students and would follow their advice blindly, 52.3% said that they would follow their advice only after confirmation by a medical doctor.

One out of three (30%) non-medical students thought that it was ok for medical students to diagnose a disease in the absence of a certified medical practitioner while 17.3% thought that it was alright for a medical student to treat an illness without any supervision.

Alarmingly it was also seen that 58.5% (n = 162) of the non-medical students had also prescribed medicines independently. The most common reasons for this were 'previous experience' (88.3%) and 'the problem being too trivial' (35.8%). This is shown in table 1 alongside the reasons given by medical students. The commonest medicines prescribed by these non-medical students were pain killers (85.2%), anti-pyretics (46.9%) and anti-allergics (42.6%) and these are shown in table 2.

Although there was no difference in the prescription rates of medical and non-medical students (p = 0.44), medical students were 3.53 times more likely to prescribe antibiotics (CI = 2.066–6.024) and 2.28 times more likely to prescribe anti-pyretics than non-medical students (CI = 1.458–3.584).

## Discussion

The issue of non-doctor prescription of medicines has not been studied enough and to our knowledge an article focusing on the prescription of medicines by medical students in particular has never been published. However there has been tremendous research on the issue of self medication which in many ways has similar implications as non-doctor prescription of medicines. However, we consider the inappropriate prescription of medicines to others as the 'bigger evil'. It puts others and society in a greater danger than self medication alone. The following discussion focuses on the prescription of medicines by non-doctors, particularly medical students. As no similar study was found in this regard it was not possible to comment upon the practices in other parts of the world.

It was very distressing to find out that every other medical student had independently prescribed medicines to someone. We had expected that a large number would be doing so but the fact that so many of them were doing it was surprising. This implies that thousands of medical students are prescribing medicines in Karachi alone. We doubt that the rates would be much different in other parts of the country however, this needs to be studied. It was overwhelming to find that non-medical students, those with an even lesser amount of knowledge regarding the complex nature of drugs, were prescribing medicines at an equal rate also. This shows that the total number of non-qualified people prescribing medicines in Pakistan, and most probably in this region, is tremendously high. This is a very serious issue.

Prescription of medicines by non-doctors is an issue of grave concern and similar to self medication it can lead to a multitude of problems including the global emergence of Multi- Drug Resistant pathogens [9], drug dependence and addiction [10], masking of malignant and potentially fatal diseases [11], hazard of misdiagnosis[12], problems relating to over and under dosaging [13], drug interactions [14] and tragedies relating to the side effect profile of specific drugs[15].

In our study the most common medicines prescribed were analgesics, antipyretics, anti-allergics and antibiotics. This can have serious implications to the individual in the form of masking of serious illnesses by analgesics and antipyretics. It could also lead to devastating effects on society especially if resilient endemic infectious diseases such as tuberculosis are harbored and treatment is delayed while symptoms are controlled by inappropriate medicines.

The issue of anti microbial resistance is one of the greatest challenges of modern times [16]. Much research and effort has been done to limit this growing menace [17]. Drug resistance is known to develop when antibiotics are taken in inappropriate doses or for inappropriate lengths of time [17]. It can also develop if the choice of antibiotics in inappropriate [17]. This study has shown that medical students prescribe antibiotics 3.5 times more than non-medical students and it has been previously demonstrated that medical students are not skilled enough to make the appropriate choice [8]. There is no doubt that non-medical students are also not knowledgeable enough to prescribe antibiotics. In this era of globalization, where intercontinental travel is rampant, the development of anti-microbial resistance in one city can have devastating effects all around the world. Hence this is not only a local concern but is an issue of great international importance.

The great respect that exists for medical professionals in society has given a helping hand to this issue. Many of our respondents stated that they would follow the advice of a medical student without consulting a doctor and many more of them said that it is alright for medical students to diagnose and treat illnesses. Our sample was derived from a highly educated slice of society and if this is what they think then it is very disturbing to imagine what the uneducated 70% of the population thinks about this. This should be a topic for further research.

There is no doubt in the fact that the issue of illegal drug prescription is a major concern and should not be neglected anymore. This situation needs to be stopped before it escalates into an insurmountable feat. A holistic multidisciplinary approach should be taken to combat this growing hidden problem. Based on the findings of our study and that of previous studies we propose four areas of intervention that should be studied and implemented as soon as possible.

First of all strict rules and regulations need to be put in place to prevent pharmacy shop owners from selling non-over the counter drugs without a doctors prescription. This is a common feature in many developing countries[6] and should not be neglected anymore. It has been shown that easy access to pharmaceuticals is a determinant for self medication [4]. Why pharmacists do not adhere to the rules should be researched further as there is paucity of literature in this regard. However, it is widely known that In Pakistan the records of pharmacy shop owners are never checked. It is up to the pharmacies own sense of ethical practice whether to sells drugs on a prescription only basis or not. Considering the fact that more sales would lead to a greater profit and in a developing country like Pakistan where the earnings by the common man are already so low and competition with other similar stores is so fierce no one would willingly give up a huge bulk of their profit. The problem is that even if they do follow the rules, the pharmacist a few shops away from them is not and thus all the customers will go to him as a matter of convenience. Another problem is that many pharmacies are not registered and thus definitely not regulated [18]. Majority of the people do not even know whether the pharmacy they go to is licensed or not [19]. A system of checks and balances should be made and brought into practice in order to ensure adequate implementation of the rules.

Secondly, there needs to be a system of similar rules and regulations to prevent robust advertising by pharmaceutical companies of their products. This includes aggressive advertising and un-ethical marketing. At present there are many weaknesses in the drug policies of Pakistan, ranging from weaknesses in the legislation to inadequate implementation of the law leading to unchecked marketing practices and the production of substandard and spurious medicines [18]. Even though direct to consumer advertising is prohibited in Pakistan there are lapses in the implementation of this law, which need to be reported and addressed. At present there is no mechanism to monitor the drug promotional campaigns by the pharmaceutical industry in Pakistan [20]. This is important as advertising directly influences the self medication practices of the people. It has been shown that familiarity with medicines leads to higher rates of self medication [4]. In a recent study it was seen that the majority of college students used at least one of the advertised products, without discussing this with their physicians[21].

Thirdly awareness regarding this issue needs to be created. The general public including non-medical students can be approached via awareness programs and/or by the media. They should be told about the serious hazardous consequences of taking medicines without a doctor's approval. For medical students, this issue needs to be addressed in the medical curriculum of medical colleges. Medical ethics should start as early as the first year as it was seen in our study that majority of students who prescribed these medicines belonged to the junior years. Many students thought that it was alright for them to diagnose and treat medical illness without the supervision of a medical doctor. This form of attitude needs to be eliminated. Students should also be taught on how to turn down a person asking for a prescription as majority of medical students said that they were asked to prescribe the medicine and conversely majority of the non-medical students said that they had indeed asked the medical student to prescribe them a drug. The effectiveness of teaching of medical ethics early on is a proven intervention [22] but sadly not many institutions in Pakistan practice this [23].

The removal of the 'barriers to health care' is the fourth intervention we propose. The availability of hassle free quality health care at an affordable price is the right of every individual and this should be protected by the state. In a poor country like Pakistan where it is so difficult to obtain quality health care the common man has no choice but to approach other avenues for the treatment of their problems. A previous study revealed that households with a lower income are more likely to seek alternate avenues for medical treatment[19]. An understanding of the exact reasons as to why people choose alternate sources of medication needs to be further studied [5].

There are a few limitations of this study that need to be taken into account. Firstly the findings of our study were based on data collected by a self reported questionnaire; hence the practices regarding drug prescription were subjectively explored. This could lead to under-reporting of the problem as social desirability bias is a common problem with this type of questionnaires. Objective studies in this regard should be carried out although they would require a great many resources, which was difficult for us to gather. The second limitation of this study lies in the sampling method. Although the sites were representative, we had taken a convenient sample of participants from these sites. This sampling method is inferior to probability sampling in its representativeness to the population. However, we feel that as the size of our sample was large its generalization to other such populations is possible. Even though the findings cannot be as accurate as those of a probability sample, we are confident that they are not far from the true findings.

## Conclusion

A great many medical and non-medical students are prescribing medicines to others and this can lead to a multitude of serious problems some of which will have global ramifications. A holistic approach needs to be taken to intervene and stop this issue from escalating. We have highlighted areas for intervention and further research, public health professionals and health policy makers should take these into account. This issue can no longer be ignored.

## Competing interests

The authors declare that they have no competing interests.

## Authors' contributions

SNZ conceived of the study, participated in its design, coordinated the processes, analyzed and interpreted the data and contributed to the writing and reviewing of the manuscript. RS, SW, FAI and SS helped in the design of the study, in data collection, drafting of the manuscript and intellectually reviewing it. All authors read and approved the final manuscript.

## Pre-publication history

The pre-publication history for this paper can be accessed here:



## Supplementary Material

Additional file 1 Questionnaire. The questionnaires used in the study. There are two separate questionnaires (one after the other) which were used for medical students and non-medical students respectively. Click here for file
