# Supplementary material for: Prescription of medicines by medical students of Karachi, Pakistan: A cross-sectional study
Source: BMC Public Health. 2008 May 19;8:162. doi: 10.1186/1471-2458-8-162 (PMC2408580; doi:10.1186/1471-2458-8-162)
Supplement: Additional file 1 — Questionnaire. The questionnaires used in the study. There are two separate questionnaires (one after the other) which were used for medical students and non-medical students respectively. [file 1471-2458-8-162-S1.doc]

Questionnaire for Medical Students. Sr no. ________

| NO | Variable | Response with codes |
| --- | --- | --- |
| **1.** | Institution |  |
| **2.** | Year of studies in this institution |  |
| **3.** | Age |  |
| **4.** | Gender | 1. Male  2. Female |
| **5.** | Area of residence? |  |
| **6.** | Religion | 1. Muslim  2. Christian  3. Hindu  4. Zoroastrian  5. Others (Please specify) ___________________ |
| **7.** | Mother tongue | 1. Muhajir/urdu speaking  2. Sindhi  3. Pathan  4. Baluchi  5. Punjabi  6. Memon  7. Others (Please specify) _______________ |
| **8.** | Monthly household income | 1. < 10,000  2. 10,000 -25,000  3. 25,000 – 50,000  4. > 50, 000 |
| **9.** | Are you suffering from any long term medical condition? (If yes please specify) | 1. Yes (Please specify)  ___________________  2. No |
| **10.** | Have you ever prescribed/advised anyone to take some medicine?  (If no then skip to question number 26) | 1. Yes  2. No |
| **11.** | Why did you prescribe these medicines? | 1. Previous experience with same  kind of illness  2. Cost of consultation  3. Urgency of problem  4. Found information related to the  illness on the internet or saw it on  TV etc  5. The problem was too trivial to go to  a doctor.  6. I knew everything about the illness  7. Others (please specify) _________ |
| **12.** | Which medicines have you prescribed? | 1. Pain killers  2. Fever-relieving medication  3. Anti-allergy  4. Antibiotics  5. Birth control pills  6. Sleeping pills  7. Tonics (for fitness)  8. Vitamins  9. Pills for indigestion  10. Herbal/homeopathic  11. others (please specify)  ____________________ |
| **13.** | How often? | 1. Once  2. Seldom (2-3 times a year)  3. Sometimes(every few months)  4. Often (every few weeks)  5. Always |
| **14.** | Did you volunteer this advice or were you asked? | 1. I volunteered it  2. I was asked |
| **15.** | Do you think it is okay for you to prescribe medicines to someone? | 1. Yes  2. No |
| **16.** | Do you think it is okay for you to prescribe medicines to someone for mild conditions if you are fully aware of his illness? | 1. Yes  2. No |
| **17.** | Do you think taking medicines without consulting a certified medical practitioner could be harmful? | 1. Yes  2. No |
| **18.** | Do you think it is okay for medical students to **diagnose** a medical illness, in the absence of a certified medical practitioner | 1. Yes  2. No  3. Don’t know |
| **19.** | Do you think it is okay for medical students to **treat** patients with medication in the absence of a certified medical practitioner? | 1. Yes  2. No  3. Don’t know |

Questionnaire for non-medical students. Sr. no. _________

| No. | Variable | Response |
| --- | --- | --- |
| **1.** | Institution |  |
| **2.** | Year of studies in this institution |  |
| **3.** | Age |  |
| **4.** | Gender | 1. Male  2. Female |
| **5.** | Area of residence? |  |
| **6.** | Religion | 1. Muslim  2. Christian  3. Hindu  4. Zoroastrian  5. Others (Please specify) ___________________ |
| **7.** | Mother tongue | 1. Muhajir/urdu speaking  2. Sindhi  3. Pathan  4. Baluchi  5. Punjabi  6. Memon  7. Others (Please specify) _______________ |
| **8.** | Monthly household income | 1. < 10,000  2. 10,000 -25,000  3. 25,000 – 50,000  4. > 50, 000 |
| **9.** | Are you suffering from any long term medical condition? (If yes please specify) | 1. Yes (Please specify)  ___________________  2. No |
| **10.** | Have you ever consulted medical students (friend, relative, acquaintance or stranger) with regard to a medical/health-related problem? (If no then skip to question number 24) | 1. Yes  2. No |
| **11.** | How often? | 1. Once  2. Seldom (2-3 times a year)  3. Sometimes(once every few months)  4. Often (once every few weeks)  5. Always |
| **12.** | Did you consult a certified medical practitioner with regard to the problem after consulting with the medical student? | 1. Yes  2. No  3. Sometimes |
| **13.** | Has a medical student ever independently prescribed or given you advice to take some medicine  (if no then skip to question number 26) | 1. Yes  2. No |
| **14.** | Did you seek this prescription or was it offered? | 1. I asked for it  2. The student volunteered to give it |
| **15.** | Would you follow the advice given by medical students | 1. Yes  2. No  3. Only after confirming with a doctor |
| **16.** | Have you ever prescribed/advised anyone to take some medicine? (If no then skip to question number 31 ) | 1. Yes  2. No |
| **17.** | Which medicines have you prescribed? | 1. Pain killers  2. Fever-relieving medication  3. Anti-allergy  4. Antibiotics  5. Birth control pills  6. Sleeping pills  7. Tonics (for fitness)  8. Vitamins  9. Pills for indigestion  10. Herbal/homeopathic  11. others (please specify)  ____________________ |
| **18.** | Why did you prescribe these medicines? | 1. Previous experience with same  kind of illness  2. Cost of consultation  3. Urgency of problem  4. Found information related to the  illness on the internet or saw it on  TV etc  5. The problem was too trivial to go to  a doctor.  6. I knew everything about the illness  7. Others (please specify) _________ |
| **19.** | How often? | 1. Once  2. Seldom (2-3 times a year)  3. Sometimes(once every few months)  4. Often (once every few weeks)  5. Always |
| **20.** | Do you think taking medicines without consulting a certified medical practitioner could be harmful? | 1. Yes  2. No |
| **21.** | Do you think it is okay for medical students to **diagnose** a medical illness, in the absence of a certified medical practitioner | 1. Yes  2. No  3. Don’t know |
| **22.** | Do you think it is okay for medical students to **treat** patients with medication in the absence of a certified medical practitioner? | 1. Yes  2. No  3. Don’t know |
